# Surface antigen expression on peripheral blood monocytes in women with gynecologic malignancies

**DOI:** 10.1186/s12885-015-1136-x

**Published:** 2015-03-15

**Authors:** Maciej Jóźwik, Osazee E Okungbowa, Alina Lipska, Marcin Jóźwik, Marzena Smoktunowicz, Andrzej Semczuk, Michał Jóźwik, Piotr Radziwon

**Affiliations:** 1 Department of Gynecology and Gynecologic Oncology, Medical University of Białystok, Skłodowskiej 24 A, 15-276 Białystok, Poland; 2 Regional Center for Transfusion Medicine, Białystok, Poland; 3 Department of Gynecology and Obstetrics, Faculty of Medicine, University of Warmia and Mazury, Olsztyn, Poland; 4 IInd Department of Gynecology, Lublin Medical University, Lublin, Poland; 5 Department of Reproductive Health, National Research Institute of Mother and Child, Warsaw, Poland; 6 Medical Institute, State Higher School of Computer Science and Business Administration, Łomża, Poland; 7 Department of Hematology, Medical University of Białystok, Białystok, Poland

**Keywords:** Cervical cancer, Endometrial cancer, Monocytes, Ovarian cancer, Surface antigens

## Abstract

**Background:**

Of many specialized blood cells, monocytes are gaining increasing attention for their role in neoplastic disorders. The purpose of the present investigation was to determine the expression of selected peripheral blood monocyte surface antigens in cases of cervical, endometrial, and ovarian cancers. In addition, our aim was to validate the diagnostic value of two artificial coefficients recently proposed for the diagnosis of gynecologic malignancies: Neutrophil to Lymphocyte Ratio (NLR), and Multiplication of Neutrophil and Monocyte Counts (MNM).

**Methods:**

We studied 69 white Caucasian women with histopathologic confirmation of endometrial (N = 42), cervical (N = 13), and ovarian (N = 14) cancers. Reference Group I were women suspected of cancer but histologically nullified (N = 20), and Group II were healthy blood donors (N = 23). Expression of CD11a, CD11b, CD11c, CD16, CD54 (ICAM-1), CD62 L (L-selectin), CD64, and HLA-DR was measured with immunofluorescence in a flow cytometer.

**Results:**

CD54 expression increased by ≥35.6% (p < 0.001) whilst HLA-DR decreased by ≥10.8% (p < 0.001) in all cancer subgroups and Group I as compared to blood donors. A correlation (p < 0.05) between CD54 and CD62 L was stronger in all cancers studied than in healthy subjects. There was no difference in the NLR values between any of these subgroups. Moreover, we observed an increase in MNM parameter in cases of cervical and endometrial cancer and in the Reference Group I.

**Conclusions:**

In the studied gynecologic malignancies, CD54 expression on peripheral blood monocytes is enhanced, indicating a higher transmigrational potential present in such patients, and HLA-DR expression diminished, indicating a decreased readiness of the immune system to recognize foreign antigens. The more pronounced correlation for the expression of CD54 and CD62 L in cancer suggests that monocytes uptake from the bloodstream and their local adhesion increase the pool of tumor-associated macrophages. This study challenged the suggested credibility and usefulness of the artificial parameters of MNM and NLR for the differential diagnosis of gynecologic malignancies.

## Background

The immune system integrates the function of the macroorganism in health and disease. Of many specialized blood cells, monocytes are gaining increasing attention for their role in neoplastic disorders for a variety of reasons. As emphasized by Hanahan and Weinberg, the biology of malignant tumors is heterotypic, i.e. they contain not only malignant cells, but also many non-malignant cells of immune character [1]. The latter include tumor-associated macrophages (TAMs). By evoking local inflammatory response and cytokine production, the innate cells of the immune system are thought to participate in tumor growth and the disease progression [2].

Numerous studies have indicated that an increase in the number of TAMs in the tumor tissue is an unfavorable predictive factor for survival in breast, prostate, ovarian and cervical cancers [3]. Whether TAMs in the specific clinical situation play a protective role against the host tissues or fulfill a procancerogenic role is determined by an interplay of environmental factors, the degree of TAMs activation, current stage of tumor’s development, and localization of TAM infiltration within the tumor mass [4-6]. Cytokines, chemokines, and growth factors produced by the tumor influence TAMs, which in return adapt to this coexistence with the secretion of matrix proteases and growth factors, suppression of acquired immunity, and angiogenesis modulation [7]. Interestingly, TAMs are known to be able to synthetize at least 3 angiostatic and 14 angiogenic factors, including angiogenin and vascular endothelial growth factor [8]. Chemokines and other angiogenic cytokines produced by TAMs are responsible for the locally increased density of microcirculation and increased vascular permeability, stabilization of vascular structure, chemotactic attraction of monocytes, and increase in the infiltration of tumor by macrophages [8]. The latter two activities associated with monocytes and macrophages continuously sustain the inflow of blood cells and maintain the interaction: TAMs - cancer cells.

Taking the above into consideration, the present work was undertaken to determine the expression of selected surface monocyte antigens (Ags) in cases of cervical, endometrial, and ovarian cancers. The Ags under study were: CD11a, CD11b, CD11c, CD16, CD54 (or ICAM-I), CD62 L (or L-selectin), CD64, and HLA-DR. The selection of these Ags was based on their ascribed important functions in the immune system [9-12]. Specifically, 4 Ags: CD11a, CD11b, CD54, and CD62 L were chosen to provide insight into cellular adhesion and monocyte-endothelial transmigration, or extravasation, and the other 4, into phagocytosis, cytotoxic reaction and presentation of foreign Ags. In addition, our secondary aim was to validate the diagnostic value of two artificial ratios recently proposed for the diagnosis of gynecologic malignancies: Neutrophil to Lymphocyte Ratio (NLR), and Multiplication of Neutrophil and Monocyte Counts (MNM) [13,14].

## Methods

### Patients

This study was conducted in accordance with the principles of the World Medical Association’s Declaration of Helsinki, the International Conference on Harmonisation Guideline for Good Clinical Practice, and the laws and regulations of Poland. All participants were white Caucasians and gave their written informed consent for the study; the Bioethics Committee of the Medical University of Białystok having earlier approved its protocol (Opinion No. RI-003/43/2006).

**Figure 1:**
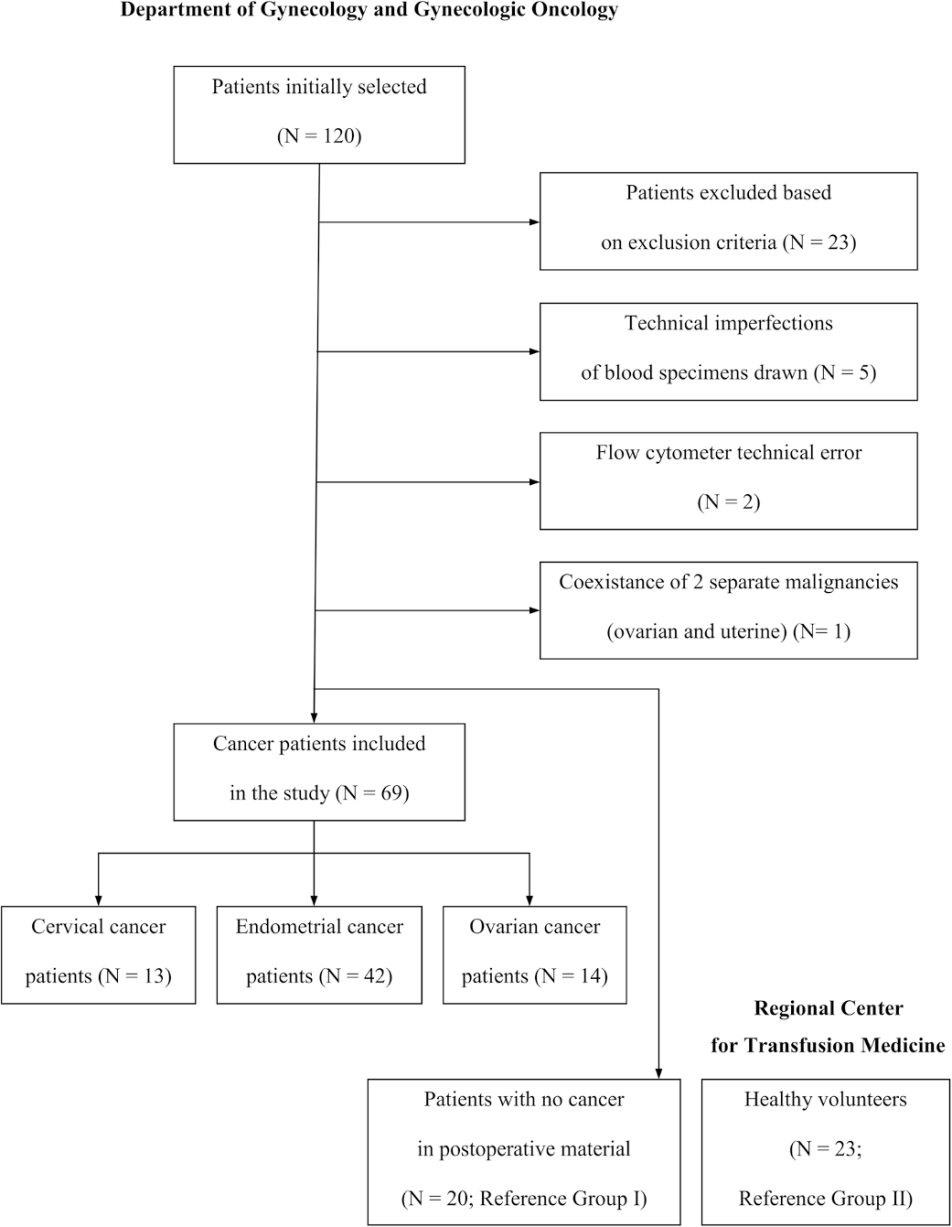
**Flow chart for the study.**

Peripheral blood from antecubital vein (10 mL) was obtained from women suspected of invasive gynecologic malignancy, admitted to the Department of Gynecology and Gynecologic Oncology, Medical University of Białystok, from April 2006 to August 2009. Preselection covered 120 patients (Figure 1). Exclusion criteria were as follows: prior treatment with chemotherapy or hormone therapy; receipt of any blood preparation over the period of 8 weeks prior to study, coexistence of malignancy other than genital tract tumor (especially malignancies of blood), stage IA1 cervical cancer (minimal microscopic stromal invasion), absence in postoperative material of uterine malignancy found earlier from dilation and curettage biopsy, adenocarcinoma *in polipo*, histopathologic diagnosis of ovarian borderline malignancy, administration of drugs modulating the immune system, any apparent acute or chronic inflammation on the body, and allergies or autoimmune disorders.

There were two reference groups. Group I (N = 20) consisted of women whose postoperative material was confirmed by anatomic pathology to be cancer free. These patients were found to have: benign ovarian tumor - in 8 cases; cervical intraepithelial neoplasia or lack of marked cervical pathology - 5; simple endometrial hyperplasia - 3; chronic inflammation: tuberculosis - 1, colitis - 1, salpingitis - 1; and endometriosis - 1. Group II (N = 23) consisted of women who were healthy blood donors. In a detailed self-reported survey of 33 questions, they declared that they were in good general health. All of them were verified for being negative for hepatitis B, hepatitis C, and HIV-1 and -2 viruses. An additional criterion for inclusion as reference was the result of sedimentation rate (SR) not higher than 10 mm • h^−1^.

Height and weight were measured using professional scale (Lublin Scale Factory, Lublin, Poland). Body mass index (BMI) was calculated in accordance with the generally accepted formula as the individual’s body mass (kg) divided by the square of their height (m).

Based on menstrual patterns history, each woman was ascribed either premenopausal, perimenopausal or postmenopausal status.

At time of surgery, material collected was immediately fixed in a 10% buffered formalin, embedded in paraffin and stained with hematoxylin and eosin for light microscopy. The histopathologic diagnosis included histological tumor type and grading according to the World Health Organization staging system and Kurman et al., respectively [15,16]. Clinical staging was done according to FIGO classification of uterine and cervical carcinomas from 2009 and of ovarian carcinomas from 1990 [17,18].

### Sample collection

Blood for the study was aseptically drawn during routine preoperative sampling, thus minimizing pain and discomfort for the patients. The following measurements were made: Ag surface expression on monocytes, leukocytosis and differential white blood cells count, C-reactive protein (CRP) concentration, and SR.

For the determination of Ag expression on monocytes and full differential blood count, blood was collected into sterile 2-mL BD Vacutainer™ K3E tubes containing K_3_EDTA as anticoagulant (catalog number 367836; Becton, Dickinson and Company (BD), Plymouth, UK). To determine the concentration of CRP, blood was collected into sterile 4-mL BD Vacutainer™ tubes (catalog number 369032). For SR determination, blood was collected into sterile 5-mL Seditainer BD™ tubes (catalog number 366674).

### Sample analysis

The SR measurement was done by a standard reading of precipitation of the meniscus of blood after 60 minutes. Other laboratory determinations were run on fully automated analyzers continuously maintained in readiness to use, undergoing regular service and operated in accordance with manufacturer’s recommendations. Full blood cell count with differential was determined using a hematological analyzer Pentra 80 (ABX Diagnostics, Montpellier, France). The CRP concentration was measured using an immunochemical analyzer ARCHITECT^®^*i*2000 SR from Abbott Diagnostics (Abbott Park, IL, USA).

**Figure 2:**
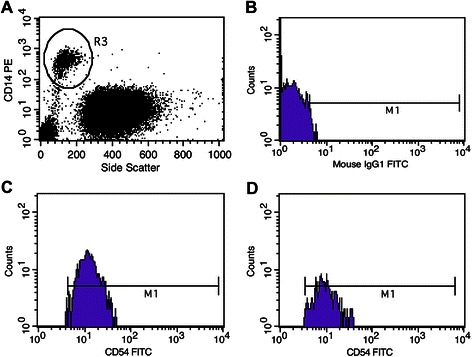
**Immunofluorescence scattergrams from flow cytometer demonstrating. A** - CD14^+^ cells (monocytes) cluster outlined from other white blood cells, **B** - negative control, and **C** - CD14^+^CD54^+^ population, all from an endometrial cancer patient. **D** - CD14^+^CD54^+^ population in a patient from Group II (healthy women). M1 - gate set on positive cells.

Monocyte surface Ag expression was studied with the immunofluorescence method in a flow cytometer FACSCalibur™ (Becton Dickinson, Franklin Lakes, NJ, USA) using antibodies (Abs) from Dako Denmark A/S (Glostrup, Denmark). The final result was expressed as a percentage of CD14^+^monocytes showing positive expression for each of the tested surface Ags. Thus, the first stage of the proceedings was to label monocytes by staining CD14 Ags on their surface with one of two CD14 Abs (catalog numbers: R0864 and F0844). In order to eliminate the influence of autofluorescence, negative controls used isotypically appropriate Abs (catalog numbers: X0927 and X0928). The following fluorochrome-labeled monoclonal Abs directed against the Ags under study were used: CD11a FITC (catalog number: F0712), CD11b PE (catalog number: R0841), CD11c FITC (catalog number: F0713), CD16 PE (catalog number R 7012), CD54 FITC (catalog number: F7143), CD62L FITC (catalog number: F7085), CD64 PE (catalog number: R7219), HLA-DR PE (catalog number: EP-R7267). Representative examples of a scattered cytogram of negative control and cytohistograms with log scale of positively labeled cells from an endometrial cancer patient and a healthy woman are presented in Figure 2.

There are two alternative forms of CD16: a transmembrane form alpha expressed by NK cells and monocyte-macrophages, and a glycosylphosphatidylinositol-linked form beta present on neutrophils [12]. In other words, the present work determined surface monocyte expression of CD16-alpha.

### Calculations and statistical analysis

The ratios NLR and MNM were calculated as initially described [13,14]: NLR - index expressed as an absolute neutrophil to lymphocytes ratio (in 1 μL of blood); MNM - index obtained by multiplying neutrophil counts per μL of blood by monocyte counts per μL of blood, and then divide by 10,000.

The distribution of particular Ags data was verified for its agreement with normal distribution using Shapiro-Wilk and Lilliefors tests. This analysis proved that approximately 80% of results were consistent with the normal distribution pattern whereas circa 20% was different from normal. Therefore, data in absolute values are expressed as both means ± standard deviation (SD) and medians with minimum-maximum range. All the differences were tested with Mann–Whitney *U* test. The relationships of staging and grading with laboratory data were verified with one-way analysis of variance (ANOVA). Verifications of other possible relationships were performed with Spearman correlation. The statistical package used was *STATISTICA*™ 10.0 (StatSoft^®^, Tulsa, OK, USA). p-Value < 0.05 was considered statistically significant.

## Results

Of the 69 women finally included in the study group, 42 had the histopathologic diagnosis of endometrioid adenocarcinoma of the endometrium, 13 - squamous cell carcinoma of the cervix, and 14 - epithelial ovarian cancer. The histological types of ovarian cancer were: serous adenocarcinoma (N = 7), endometrioid (N = 5), and clear cell carcinoma (N = 2). Table 1 presents the clinical characteristics of the two reference groups and the study group with division into cancer subgroups. As expected, healthy blood donors forming Group II were the youngest and with the lowest proportion of subjects after menopause. On the average, endometrial cancer patients were obese.

**Table 1:** **Clinical characteristics of the studied women**

Clinical Data	Endometrial Cancer	Cervical Cancer	Ovarian Cancer	Group I	Group II
	(N = 42)	(N = 13)	(N = 14)	(N = 20)	(N = 23)
Age (years)	62.52 ± 9.81;	48.77 ± 12.94;	53.21 ± 6.68;	53.42 ± 14.51;	42.52 ± 9.18;
	61.50 ***	49.00	54.00 ***	51.50 **	45.00
	(44–83)	(27–66)	(40–65)	(24–78)	(25–59)
Gravidity	2.88 ± 2.20;	1.54 ± 1.33;	2.79 ± 2.04;	2.10 ± 2.40;	Insufficient data
	2.50;	1.00;	2.50;	2.00;	
	(0–13)	(0–5)	(0–8)	(0–10)	
Parity	2.69 ± 1.96;	1.15 ± 0.90;	2.57 ± 1.74;	1.85 ± 2.06;	Insufficient data
	2.00;	1.00;	2.00;	2.00;	
	(0–12)	(0–3)	(0–7)	(0–8)	
Body mass (kg)	87.13 ± 20.56;	62.46 ± 11.21;	65.09 ± 10.21;	71.30 ± 17.34;	Insufficient data
	85.00	60.00	69.00	68.50	
	(45–130)	(46–80)	(48–78)	(47–120)	
Height (cm)	159.68 ± 6.73;	160.54 ± 5.56;	161.43 ± 4.29;	164.35 ± 5.11;	Insufficient data
	160.00	162.00	160.00	164.00	
	(135–172)	(152–172)	(157–170)	(156–174)	
BMI (kg • (m^2^)^-1^)	34.14 ± 7.82;	24.38 ± 4.99;	24.97 ± 3.81;	26.37 ± 6.20;	Insufficient data
	31.94	22.77	26.43	24.86	
	(20.31-50.78)	(15.55-33.73)	(18.28-28.92)	(18.83-4.62)	
Percentage of subjects after menopause (%)	90.48 ***	46.15	71.43 **	60.00 *	26.09

Group I - women operated but had no malignancy in postoperative histopathologic examination, Group II - healthy women. BMI - Body Mass Index. Data are shown as: mean ± 1 SD, median (minimum-maximum). Asterisks indicate statistically significant differences in Mann–Whitney *U* test: * - p < 0.05; ** - p < 0.01; *** - p < 0.001, in comparison to Group II.

Breakdown by stage for endometrial cancer i.e.: 61.9% (n = 26) stage I, 19.0% (n = 8) stage II, 16.7% (n = 7) stage III, and 2.4% (n =1) stage IV. Cervical cancer was: 38.5% (n = 5) stage IA2, 30.8% (n = 4) stage IB, and 15.4% (n = 2) stage IIA (2 patients were transfered to another oncological center). Ovarian cancer was: 21.4% (n = 3) stage II, 64.3% (n = 9) stage III, and 14.3% (n = 2) stage IV. Endometrial cancer grading was: 14.3% (n = 6) G1, 69.0% (n = 29) G2, and 16.7% (n = 7) G3. Cervical cancer grading was: 7.7% (n = 1) G1, 46.2% (n = 6) G2, and 15.4% (n = 2) G3 (in 4 cases, no grading was provided). Ovarian cancer grading was: 28.6% (n = 4) G1, 42.9% (n = 6) G2, and 28.6% (n = 4) G3.

Table 2 presents data on the expression of the 8 selected monocyte surface Ags. Two clear-cut observations were made: CD54 expression was significantly increased by ≥ 35.6% (p < 0.001) and HLA-DR expression was decreased by ≥ 10.8% (p < 0.001) in all the cancer subgroups and women operated for benign conditions as compared to healthy blood donors. For CD11a, CD11b, CD11c, and CD64, there was no particular pattern of change in the expression in health and disease.

**Table 2:** **Expression of surface antigens on peripheral blood monocytes in the studied women**

Laboratory Data	Endometrial Cancer (N = 42)	Cervical Cancer (N = 13)	Ovarian Cancer (N = 14)	Group I (N = 20)	Group II (N = 23)
CD11a (%)	99.98 ± 0.06;	99.94 ± 0.21;	99.44 ± 1.94;	99.99 ± 0.05;	99.96 ± 0.09;
	100.00	100.00	100.00	100.00	100.00
	(99.74-100.00)	(99.23-100.00)	(92.72-100.00)	(99.79-100.00)	(99.59 -100.00)
CD11b (%)	99.72 ± 0.73;	99.35 ± 1.60;	99.87 ± 0.16;	99.72 ± 0.45;	99.91 ± 0.16;
	99.87 *	99.86 *	99.93	99.93 *	100.00
	(95.28-100.00)	(94.08-100.00)	(99.40-100.00)	(98.07-100.00)	(99.47-100.00)
CD11c (%)	99.54 ± 1.36;	97.62 ± 6.74;	98.27 ± 5.87;	99.77 ± 0.37;	99.40 ± 2.37;
	100.00	99.93	100.00	99.96	100.00
	(92.49-100.00)	75.38-100.00)	(77.93-100.00)	(98.91-100.00)	(88.63-100.00)
CD16 (%)	17.86 ± 22.51;	11.99 ± 18.09;	26.19 ± 31.96;	16.99 ± 26.21;	10.64 ± 4.95;
	7.19	6.16 *	8.84	6.37 **	9.55
	(2.39-83.80)	(1.23-69.87)	(4.24-91.53)	(2.78-85.20)	(5.23-28.30)
CD54 (%)	83.89 ± 16.44;	87.27 ± 12.32;	89.86 ± 11.94;	92.40 ± 9.16;	63.82 ± 15.21;
	88.46 ***	91.46 ***	94.57 ***	97.00 ***	65.20
	(27.75-100.00)	(68.40-100.00)	(63.90-100.00)	(69.87-100.00)	(21.61-91.75)
CD62L (%)	97.64 ± 2.06;	96.51 ± 4.99;	98.80 ± 1.51;	93.69 ± 9.74;	96.56 ± 2.83;
	98.35	97.85	99.18 ***	98.23	97.01
	(93.00-100.00)	(80.38-99.54)	(94.74-100.00)	(67.94-99.86)	(85.40-99.70)
CD64 (%)	95.57 ± 9.06;	93.38 ± 7.42;	97.88 ± 4.67;	96.80 ± 5.22;	99.39 ± 1.19;
	99.16 ***	96.30 **	99.65	99.59 *	99.91
	(47.60-100.00)	(76.60-100.00)	(82.13-100.00)	(81.93-100.00)	(95.15-100.00)
HLA-DR (%)	80.94 ± 15.85;	68.50 ± 25.66;	73.98 ± 16.60;	70.22 ± 24.76;	94.98 ± 5.66;
	85.80 ***	76.60 ***	73.35 ***	79.70 ***	96.10
	(33.13-99.71)	(18.50-95.83)	(27.65-95.21)	(17.33-99.37)	(73.48-99.63)

Group I - women operated but had no malignancy in postoperative histopathologic examination, Group II - healthy women. SD - standard deviation, CD - cluster of differentiation molecule, HLA-DR - membrane receptor: Human leukocyte antigen-DR (product of *hla-dr* locus). Data are presented as: mean ± 1 SD, median (minimum-maximum). Asterisks indicate statistically significant differences in Mann–Whitney *U* test: * - p < 0.05; ** - p < 0.01; *** - p < 0.001, in comparison to Group II.

Table 3 presents interrelationships between these surface Ags. For endometrial and ovarian cancers, there was a correlation for CD11a and CD54, but not for CD11b and CD54. The correlation between CD11b and CD64 was significant for endometrial and cervical cancers coupled with healthy blood donors. The correlation between CD54 and CD62 L was statistically important for all the cancer subgroups and healthy women.

**Table 3:** **Statistically significant correlations between the 8 monocyte surface antigens under study, for the particular subgroups**

Endometrial Cancer (N = 42)	Cervical Cancer (N = 13)	Ovarian Cancer (N = 14)	Group I (N = 20)	Group II (N = 23)
CD11a vs. CD54 (r = 0.444920; p = 0.0032)	(−)	CD11a vs. CD54 (r = 0.606311; p = 0.0216)	(−)	(−)
CD11b vs. CD64 (r = 0.390416; p = 0.0106)	CD11b vs. CD64 (r = 0.556329; p = 0.0484)	(−)	(−)	CD11b vs. CD64 (r = 0.534304; p = 0.0087)
(−)	CD11b vs. CD62 L (r = 0.789189; p = 0.0014)	(−)	(−)	(−)
(−)	CD11c vs. CD64 (r = − 0.645134; p = 0.0173)	(−)	(−)	(−)
(−)	CD11c vs. HLA-DR (r = 0.672398; p = 0.0119)	(−)	CD11c vs. HLA-DR (r = 0.445866; p = 0.0488)	(−)
(−)	(−)	CD16 vs. CD62 L (r = 0.554456; p = 0.0397)	(−)	(−)
(−)	(−)	(−)	(−)	CD16 vs. HLA-DR (r = − 0.506677; p = 0.0137)
CD54 vs. CD62 L (r = 0.518139; p = 0.0005)	CD54 vs. CD62 L (r = 0.570250; p = 0.0419)	CD54 vs. CD62 L (r = 0.550661; p = 0.0413)	(−)	CD54 vs. CD62 L (r = 0.426489; p = 0.0425)
(−)	(−)	(−)	CD54 vs. HLA-DR (r = −0.476692; p = 0.0336)	(−)

Group I - women operated but had no malignancy in postoperative histopathologic examination, Group II - healthy women. r - Spearman correlation coefficient.

In the endometrial cancer group (n = 42), in Mann–Whitney *U* test, the expression of HLA-DR in cases of stage I (median 82.82%) differed significantly (p = 0.0055) from the expression for stage II (median 93.00%; not significant in comparison with other stages). The expression of HLA-DR correlated directly with staging (r = 0.3613, p = 0.0188) and grading (r = 0.3864, p = 0.0126), and inversely with CRP (r = −0.4092, p = 0.0079).

Table 4 presents data on SR, CRP concentration, and white blood cell counts and integrates them with the calculated coefficients: NLR and MNM. SR was increased (p < 0.05) in all the cancer subgroups as compared to healthy blood donors, and CRP was increased (p < 0.01) for cervical and ovarian cancers. Importantly, results from these studies showed that there was no difference in the NLR values between any of these subgroups. Moreover, we observed an increase in MNM parameter not only in cases of cervical cancer (p = 0.0057), but also in cases of endometrial cancer (p < 0.0001) and in Group I (p = 0.0077). Another important finding from Table 4 was that the monocyte count markedly rose (p < 0.001) in cases of endometrial and ovarian cancers in comparison to healthy blood donors. In other words, the pool of monocytes available for becoming TAMs increased in these patients.

**Table 4:** **Laboratory data, together with the calculated NLR and MNM coefficients, for the studied women**

Parameter	Endometrial Cancer (N = 42)	Cervical Cancer (N = 13)	Ovarian Cancer (N = 14)	Group I (N = 20)	Group II (N = 23)
SR (mm • h^-1^)	20.47 ± 14.11;	16.58 ± 16.81;	23.58 ± 12.94;	17.60 ± 20.24;	7.39 ± 1.70;
	18.00 ***	12.00 *	21.00 ***	9.00	8.00
	(2–59)	(2–62)	(5–40)	(2–70)	(4–10)
CRP (mg • L^-1^)	9.63 ± 19.16;	4.48 ± 8.68;	60.06 ± 62.42;	9.59 ± 18.94;	1.63 ± 0.85;
	3.60	0.00 **	34.00 ***	0.00	1.50
	(0.00-96.30)	(0.00-23.30)	(0.00-204.00)	(0.00-78.50)	(0.50-4.00)
Leukocytosis (10^3^ • μL^-1^)	7.94 ± 2.42;	8.64 ± 2.48;	7.16 ± 2.09;	7.02 ± 1.76;	6.10 ± 1.30;
	7.80 **	8.35 **	6.90	7.41 *	6.30
	(3.80-14.40)	(5.30-13.30)	(4.20-11.40)	(4.10-11.01)	(4.50-8.50)
Neutrophils (%)	57.40 ± 5.44;	61.38 ± 9.83;	58.21 ± 5.45;	52.64 ± 7.49;	57.39 ± 5.90;
	57.00	62.10	57.50	54.35	56.90
	(26.00-78.50)	(29.30-84.60)	(48.70-69.80)	(38.00-75.70)	(47.60-69.70)
Lymphocytes (%)	31.51 ± 5.31;	29.33 ± 9.64;	30.66 ± 5.15;	36.80 ± 6.92;	32.33 ± 6.20;
	30.95	27.10	31.60	35.40	32.80
	(12.00-58.50)	(9.40-63.10)	(17.40-41.60)	(11.30-51.80)	(20.30-45.00)
Monocytes (%)	8.12 ± 1.46;	7.09 ± 1.35;	7.87 ± 1.45;	9.14 ± 1.87;	6.46 ± 0.76;
	7.95 ***	7.10	7.70 ***	8.60 ***	6.60
	(4.80-13.10)	(4.70-9.50)	(5.00-10.90)	(6.20-14.10)	(4.70-9.60)
NLR (no titer)	2.00 ± 0.92;	2.85 ± 2.19;	2.05 ± 0.76;	1.69 ± 1.25;	1.91 ± 0.67;
	1.82	2.31	1.84	1.65	1.77
	(0.44-6.54)	(0.46-9.00)	(1.17-4.01)	(0.73-6.70)	(1.06-3.43)
MNM (no titer)	3.11 ± 2.00;	3.04 ± 2.12;	2.44 ± 1.73;	2.62 ± 1.79;	1.43 ± 0.61;
	2.80 ***	2.30 **	1.92	2.48 **	1.42
	(0.41-11.07)	(0.00-7.63)	(0.00-6.30)	(0.63-7.90)	(0.57-2.41)

Group I - women operated but had no malignancy in postoperative histopathologic examination, Group II - healthy women. *SD* - standard deviation, *SR* - sedimentation rate, *CRP* - C-Reactive Protein, *NLR* - Neutrophil to Lymphocyte Ratio, *MNM* - Multiplication of Neutrophil and Monocyte Counts. Data are presented as: mean ± 1 SD, median (minimum-maximum). Asterisks indicate statistically significant differences in Mann–Whitney *U* test: * - p < 0.05; ** - p < 0.01; *** - p < 0.001, in comparison to Group II.

**Figure 3:**
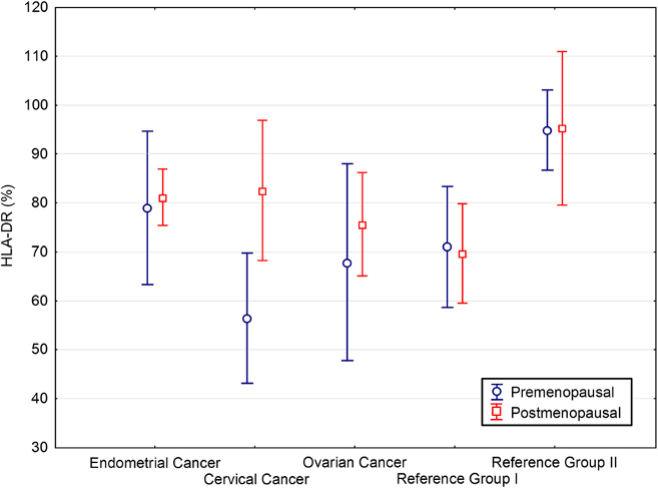
**Expression of monocyte HLA-DR surface antigen in the studied women according to their hormonal status.** Group I - women operated but had no malignancy in postoperative histopathologic examination, Group II - healthy women. Circles and squares represent means, bars represent 95% confidence intervals. For cervical cancer, the difference between premenopausal and postmenopausal women was of borderline significance (p = 0.0537).

As for the hormonal status, there were no perimenopausal subjects. The analysis of the impact of hormonal status on monocyte Ags expression suggested that postmenopausal women with cervical cancer demonstrate a higher HLA-DR expression than premenopausal women (p = 0.0537; Figure 3). No further impact of gravidity, parity, height, weight, BMI, SR, CRP, NLR, MNM, and ovarian cancer histologic types on the examined Ags was confirmed.

## Discussion

When monocytes present in the bloodstream transmigrate through the vessel wall into the tissue, they are known as macrophages. Similarly, when the migration occurs into a tumor, such monocytes are labeled TAMs. So far, there is no evidence that the processes of transmigration and colonization within the neoplasm result in immediate changes of the TAMs’ surface antigenicity. With this assumption, studies of the expression of surface Ags on monocytes derived from peripheral blood may shed light on the interplay of tissue macrophages with other cells, including neoplastic cells.

To date, this topic has not systematically been explored in cases of gynecologic malignancies. In 1989, De Jaco et al. reported briefly on a small group of gynecologic cancer patients in whom plasma levels of CD16 increased with advancing stages of the disease [19]. In 2012, Brooks et al. reported that CD16 expression on blood monocytes in 12 endometrial cancer patients was not significantly different from that observed in 10 controls [20].

The present study is the ever first systematic investigation undertaken on peripheral blood monocytes Ags in endometrial and cervical cancers and adds to the limited existing knowledge of ovarian cancer. Its principal finding is that CD54 expression is enhanced, whereas HLA-DR expression is diminished (Table 2). Thus, the CD54 molecule which facilitates leukocyte-endothelial transmigration is more abundant on monocytes, while the HLA-DR molecule responsible for the presentation of foreign peptide Ags to the immune system is less. The first finding indicates a higher transmigrational potential present in our cancer patients. The second finding can be interpreted as a decreased readiness of the immune system to recognize cancer cells. In their recent commentary, Scarlett and Conejo-Garcia argued that the crucial event driving aggressive malignant ovarian expansion is leukocyte-mediated immunosuppression, rather than loss of recognizable tumor antigens [21]. Our data on the decreased monocyte HLA-DR expression in cancer suggest that the diminished recognition of foreign antigens could be a viable option. In fact, in 2006, Gordon and Freedman demonstrated a defective Ab-dependent cytotoxicity and phagocytic function of human epithelial ovarian cancer-associated monocytes [22]. In comparison to normal blood monocytes, ascitic monocytes and blood monocytes of ovarian cancer patients exhibit decreased migration in response to chemokine ligands [23]. This is further supported by data of Loercher et al. who identified a subset of interleukin-10-producing HLA-DR-negative monocytes from ascites of ovarian cancer patients that inhibited T cells proliferation [24]. This would also be in line with a slightly but significantly decreased expression of CD64 (which is involved in phagocytosis and cytotoxic reaction) in our endometrial and cervical cancer patients. The correlation for the expression of adhesion molecules CD54 and CD62 L was more pronounced for all cancers studied than in healthy controls (Table 3), suggesting that the uptake of macrophages from the bloodstream and their local adhesion increase the pool of TAMs in the tumor. Recently, increase in CD54 expression on colorectal and pancreatic cancer cells has been shown to promote adhesion to the senescent peritoneal mesothelium [25]. CD54 mediates the initial capture of melanoma cells by polymorphonuclear leukocytes [26]. We observed a pronounced rise in CD54 expression for our studied cancers. For ovarian cancer in particular, this could relate to its facilitated ability to metastasize to the peritoneum, provided similar mechanisms promoting CD54 expression on both monocytes and neoplastic cells exist. Recent studies of mucin 2 molecule expression by human ovarian cancer cells revealed that it induces local prostaglandin PGE_2_ synthesis in both TAMs and cancer cells and the maintenance of a positive feedback between PGE_2_ synthesis and TAM polarization into clinically unbeneficial phenotype M2 accelerates cancer progression [27].

It is thought that CD54 function can be mediated via activated CD18 integrins: CD11a or CD11b [9]. From our results (Table 3), CD54 function in endometrial and ovarian cancers appears to mediate via integrin CD11a, but not CD11b.

As for NLR and MNM, there was no difference in the NLR values between any subgroups (Table 4), which has given rise to question the validity and credibility for the usefulness of this parameter, despite the limited number of observations. We confirm an increase in MNM parameter in the case of cervical cancer [14]. However, we also found MNM increases in endometrial cancer and in the Group I. The lack of MNM selectivity for cervical cancer disproves the credibility of this parameter in the diagnosis of specific gynecologic cancers. Furthermore, the 8 studied Ag molecules do not distinguish patients with gynecologic malignancies from healthy subjects, nor from benign gynecologic conditions.

We acknowledge the limitations of our study due to insufficient clinical data from healthy blood donors (which might affect our conclusions) and a limited number of observations on cervical cancer patients. Nonetheless, for the endometrial and ovarian cancer patients, we were able to demonstrate 3 simultaneous findings pointing to the biologically important phenomenon of enhanced transmigration: increased monocyte count, increased expression of monocyte CD54 Ag, and its significant correlation with the expression of another molecule participating in the transmigration, CD62 L.

Further studies on the roles of monocyte surface Ags CD54 and HLA-DR in gynecologic malignancy are warranted.

## Conclusions

In summary, our study demonstrated that, in cases of endometrial, cervical, and ovarian cancer, CD54 expression on peripheral blood monocytes is enhanced and HLA-DR expression diminished. These observations indicate both a higher transmigrational potential present in such patients and a decreased readiness of their immune system to recognize foreign antigens. This study strongly challenged the suggested credibility and usefulness of the artificial parameters of MNM and NLR for the differential diagnosis of gynecologic malignancies.

## Abbreviations

Ab: Antibody
Ag: Antigen
BMI: Body mass index
CD: Cluster of differentiation
CRP: C-reactive protein
MNM: Multiplication of neutrophil and monocyte counts
NLR: Neutrophil to lymphocyte ratio
SD: Standard deviation
SR: Sedimentation rate
TAM: Tumor-associated macrophage
